# sCLU regulates cisplatin chemosensitivity of lung cancer cells *in vivo*

**DOI:** 10.1186/s12957-015-0501-1

**Published:** 2015-02-25

**Authors:** Guoliang Ma, Hengjuan Cai, Lizhen Gao, Mei Wang, Haixia Wang

**Affiliations:** Department of Clinical Lab, People’s Hospital of Laiwu, Laiwu, China; Department of neurology, People’s Hospital of Zhangqiu, Zhangqiu, China; Department of Clinical Lab, People’s Hospital of Zhangqiu, Zhangqiu, China; Department of Blood Transfusion, The Affiliated Hospital of Weifang Medical College, Weifang, China

**Keywords:** Lung cancer, Chemotherapy, Clusterin, AKT, ERK1/2

## Abstract

**Background:**

In a previous analysis using a lung cancer cell line model, we have found that therapies directed against secreted clusterin (sCLU) and its downstream signaling targets pAkt and pERK1/2 may have the potential to enhance the efficacy of cisplatin (DDP)-based chemotherapy *in vitro*. Here, we investigated the therapies directed against sCLU on the DDP-based chemotherapy *in vivo* and explored the mechanism.

**Methods:**

Using lung cancer cell lines, A549 cells and DDP-resistant A549 cells (A549^DDP^), we determined the effect of sCLU silencing using short interfering double-stranded RNA (siRNA) on chemosensitivity in immunocompromised mice bearing A549^DDP^ tumors. We then determined the effect of sCLU overexpression via stable sCLU transfection on chemosensitivity in immunocompromised mice bearing A549 tumors. The effect of sCLU silencing or overexpression on pAkt and pERK1/2 expression and chemosensitivity *in vivo* was detected by Western blot assay.

**Results:**

The results showed sCLU silencing increased the chemosensitivity of A549^DDP^ cells to DDP *in vivo* via downregulation of pAkt and pERK1/2 expression. And sCLU overexpression decreased the chemosensitivity of A549 cells to DDP *in vivo* via upregulation of pAkt and pERK1/2 expression.

**Conclusions:**

We therefore concluded that the DDP-induced sCLU activation, which involved induction of pAkt and pERK1/2 activation that confer DDP resistance in immunocompromised mice and alteration of this balance, allows sensitization to the antitumor activity of cisplatin chemotherapy.

## Background

Lung cancer is known to be the most frequent cancer worldwide and the incidence of this epidemic disease is continuing to increase at 0.5% per year globally [1]. Because of the size and distribution of lung cancer, the cytoreductive surgery is not very effective for this disease and therefore chemotherapy and/ or radiation are the only treatments of choice. Despite major advances in patient management, chemotherapy and radiotherapy, nearly 80% of the patients still die within 1 year of diagnosis and long-term survival is obtained only in 5% to 10% of cases [1].

Cisplatin (DDP) has been the most widely used drug in first-line chemotherapy. The major obstacle in lung cancer chemotherapy is the emergence of inherent and acquired drug resistance in cancer cells [2,3]. The efficacy of chemotherapy is thus limited. To overcome this resistance, often higher doses of toxic anticancer drugs are administered to cancer patients, thus resulting in adverse side effects to healthy organs and tissues. In this regard, reversal of drug resistance is one of the most attractive ways to significantly enhance therapeutic efficacy in lung cancers.

The cytoprotective chaperone protein, clusterin, is synthesized as full-length clusterin (60 kDa) in the mitochondria and is targeted to the endoplasmic reticulum, where it is glycosylated, proteolytically cleaved into an a and b chain, and secreted into the extracellular matrix as the secreted form of clusterin (40 kDa). Clusterin protein is commonly upregulated by cytotoxic chemotherapy and radiotherapy in cancer cells and contributes to cancer cell resistance *in vitro* and in various animal models of cancer by blocking apoptosis [4]. Recent clinical trials of OGX-011, an antisense oligonucleotide specifically targeting clusterin, have shown promise when combined with chemotherapy in cancer patients [5].

Several *in vitro* studies have examined the role of clusterin in carcinogenesis, lung cancer progression, and response to chemo- and radiotherapy [6-14]. Studies performed in lung cancer cell lines and animal models showed that clusterin is upregulated after exposure to chemo- and radiotherapy [7,8,11]. A potential role proposed for the protein is cytoprotective. *In vitro*, clusterin silencing by antisense oligonucleotides (ASO) and small-interfering RNAs (siRNA) directed against clusterin mRNA in clusterin-rich lung cancer cell lines sensitized cells to chemotherapy and radiotherapy and decreased their metastatic potential [8,9,11,12,14].

We have shown secreted clusterin (sCLU) silencing directed against sCLU mRNA in sCLU-rich lung cancer cell lines sensitized cells to DDP chemotherapy *in vitro* [11]. The molecular mechanisms underlying the effect of sCLU silencing on lung cancer cell chemosensitivity is via its downstream signaling targets pAKT and pERK1/2. The current study investigated the significance of clusterin (sCLU) silencing on DDP chemosensitivity in lung cancer cell lines *in vivo* and investigated the molecular mechanisms underlying the effect of sCLU silencing.

## Methods

### Cell lines

Human lung adenocarcinoma bronchioloalveolar carcinoma A549 cells and cisplatin (DDP) resistant A549 cells (A549^DDP^) were obtained from the American Type Culture Collection (Manassas, VA, USA) and cultured at 37°C in a humidified atmosphere containing 5% CO_2_ in Ham’s F12 medium supplemented with sodium bicarbonate (2.2%, *w*/*v*), L-glutamine (0.03%, *w*/*v*), penicillin (100 units/ml), streptomycin (100 μg ml^−1^), and fetal calf serum (10%).

### Reagents

Akt (Ab-^473^) antibody (E021054-2), ERK1/2 (Ab-^202/204^) antibody (E022017-2), ERK1/2 (Phospho-Thr^202/Tyr204^) antibody (E012017-2), AKT (Phospho-Ser^473^) antibody (E011054-2) and β-actin antibody (5B7) (E12-041-3), clusterin (A-9) (sc-166907, 1:200), the enhanced chemiluminescence detection kit, and DDP were preserved in our laboratory [11]. The pCDNA3.1 and pCDNA3.1-sCLU plasmid and the sCLU-shRNA and control scrambled plasmid were also preserved in our laboratory [11].

### pCDNA3.1-sCLU and sCLU-shRNA transfection

pCDNA3.1-sCLU and its control pCDNA3.1 plasmid were transfected into the A549 cells to product stably transfected cell populations (A549**/**sCLU and A549**/**pCDNA3.1) as reported previously [11]. sCLU-shRNA and control scrambled plasmid were transfected into the A549^DDP^ cells to product stably transfected cell populations (A549^DDP^**/**sCLU-shRNA and A549^DDP^**/**shRNA) as reported previously [11].

### Subcutaneous implantation of tumor cells

A549, A549^DDP^, A549**/**sCLU, A549**/**pCDNA3.1, A549^DDP^**/**sCLU-shRNA, and A549^DDP^**/**shRNA cells were harvested from subconfluent cultures after a brief exposure to 0.25% trypsin and 0.2% EDTA. Trypsinization was stopped by adding medium containing 10% FBS. The cells were washed once in serum-free medium and resuspended in PBS. Only suspension consisting of a single cell with >90% viability was used for the injections. Cells (2 × 10^6^) in 100 μl PBS were injected s.c into the right flank on 6-week-old male nude (athymic) mice with a 27-gauge hypodermic needle, respectively. In our previous experience with this model, tumors take rate of **>**95% was obtained.

### Experimental protocol

All surgical procedures and care administered to the animals were in accordance with institutional animal ethic guidelines. Tumors were established by subcutaneous injection of 2 × 10^6^/A549 tumor cells (A549, A549^DDP^, A549**/**sCLU, A549**/**pCDNA3.1, A549^DDP^**/**sCLU-shRNA and A549^DDP^**/**shRNA, respectively) into the flanks of mice. Tumor volumes were estimated according to the formula: *p*/6× *a*^2^ × *b*, where *a* is the short axis and *b* the long axis. When tumors reached −100 mm^3^ at about 3 weeks, the mice were randomly assigned to 2 groups (each group had 8 mice): control and DDP. Mice received daily 200 μl i.p. injections of either PBS or DDP (4 mg/kg body/wt.,i.p), respectively. DDP was administered i.v. once every 3 days. The treatments lasted for 15 days during which the size of the tumors was recorded. The mice were euthanized 3 days after the last injection, and tumors were excised. Each tumor was divided into two halves, one half was fixed with 10% buffered formalin and the other stored at −80°C.

### Western blotting

Tumor tissues were excised, minced, and homogenized in protein lysate buffer. Debris was removed by centrifugation. Samples containing 40 μg of total protein were resolved on 12% polyacrylamide SDS gels and electrophoretically transferred to polyvinylidene difluoride (PVDF) membranes. The membranes were blocked with 5% skim milk and incubated with primary antibody and subsequently with an alkaline phosphatase-conjugated secondary antibody. Blots were stained with an anti-β-actin Ab to confirm that each lane contained similar amounts of homogenate.

### *In situ* detection of apoptotic cells

Tumor sections were stained with the TUNEL agent (Roche, Shanghai, China), and the TUNEL-positive cells were counted in 10 randomly selected × 400 high-power fields under microscopy. The apoptosis index was calculated according to the following formula: the number of apoptotic cells/total number of nucleated cells × 100%.

### Statistical analysis

Data were expressed as mean ± standard deviation (SD). Experiments were performed at least in triplicate. Comparisons were done with two-tailed Student’s *t* test or ANOVA. A value of *P* < 0.05 was considered statistically significant.

## Results

### Clusterin overexpression *in vivo* significantly increased the resistance of the lung cancer cells to cisplatin

**Figure 1:**
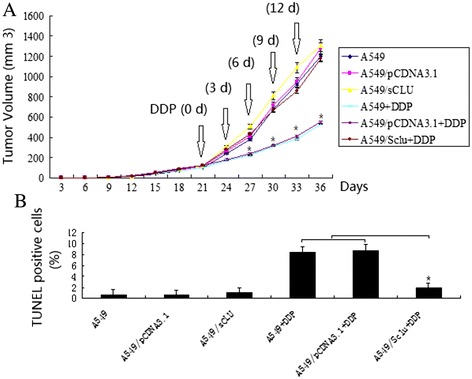
**Clusterin overexpression decreased chemotherapeutic sensitivity and inhibited apoptosis of DDP sensitive A549 cells. (A)** Cells transfected with pCDNA3.1 /sCLU or scramble pCDNA3.1 were injected subcutaneously into the right flank of nude mice. When tumors reached −100 mm^3^ in volume, the mice received daily 200 μl i.p. injections of DDP (4 mg/kg body/wt.,i.p). DDP was administered i.v. once every 3 days. The treatments lasted for 15 days during which the volume of tumors was recorded,*P < 0.05(vs pCDNA3.1 /sCLU + DDP) (Student’s *t* test). **(B)** Tumor sections were stained with TUNEL agent to visualize apoptotic cells. * *P* < 0.05 (Student’s *t* test).

Based on the *in vitro* experiment of clusterin in the cisplatin resistance of lung cancers [11], we further examined if clusterin expression affects the cisplatin sensitivity *in vivo*; A549, A549**/**sCLU, and A549**/**pcDNA3.1 cells were injected subcutaneously into the right flank of nude mice. Cisplatin could significantly inhibit the tumor growth in mice injected with A549**/**pCDNA3.1 scramble cells and A549 cells compared to the mice injected with A549**/**sCLU cells (Figure 1A). As shown in Figure 1A, the A549 tumors of mice treated with DDP only reached 530 ± 18.6 mm^3^ in volume 36 days after treatment, which was significantly smaller compared to A549**/**sCLU cells (1184.4 ± 102.6 mm^3^) in volume 36 days after DDP treatment (*P* < 0.05). Clusterin overexpression alone showed no significant growth inhibition compared to the control group.

**Figure 2:**
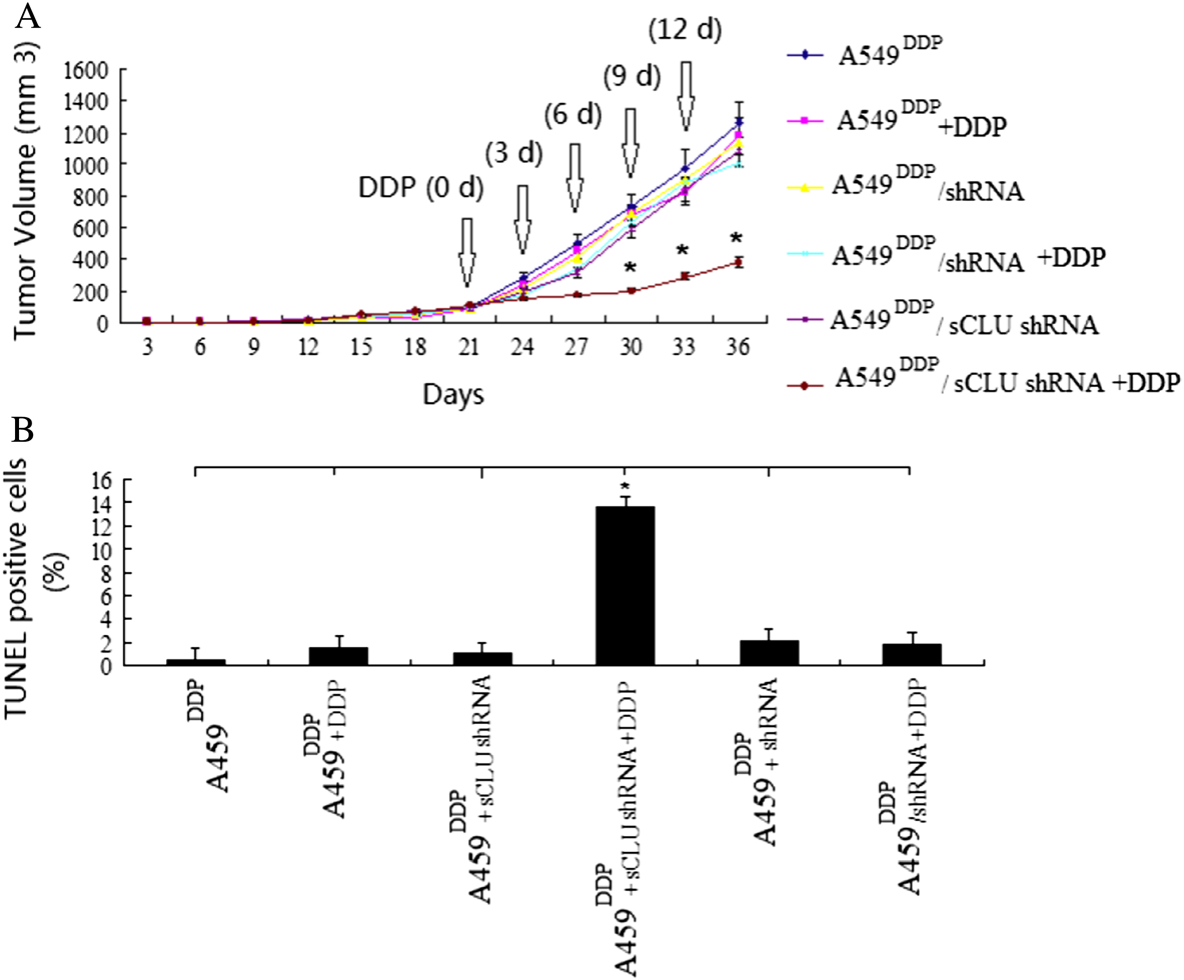
**Clusterin silencing increased chemotherapeutic sensitivity and promoted apoptosis of DDP resistant A549 cells. (A)** A549^DDP^ cells transfected with sCLU shRNA or scramble shRNA were injected subcutaneously into the right flank of nude mice. When tumors reached −100 mm^3^ in volume, the mice received daily 200 μl i.p. injections of DDP (4 mg/kg body/wt.,i.p). DDP was administered i.v. once every 3 days. The treatments lasted for 15 days during which the volume of tumors was recorded,*P < 0.05 (Student’s *t* test). **(B)** Tumor sections were stained with TUNEL agent to visualize apoptotic cells.^*^ *P* < 0.05 (Student’s *t* test).

Tumor sections prepared from the three groups were stained with the TUNEL agent to detect apoptotic cells. The results in Figure 2B showed that there were more apoptotic cells in tumors (A549 and A549**/**pCDNA3.1) treated with DDP compared with the control tumors. There were few apoptotic cells in tumors (A549**/**sCLU) treated with DDP, compared with the control tumors (Figure 1B, *P* < 0.05). Clusterin overexpression alone showed no significantly increased apoptosis compared to the control group.

### Clusterin silencing *in vivo* significantly decreased the resistance of the lung cancer cells to cisplatin

To compare the *in vivo* antitumor activities of clusterin silencing and DDP monotherapies, the two therapies were evaluated in an A549^DDP^ mouse xenograft model. We found no significant decreases in tumor volume with clusterin silencing and DDP monotherapies (Figure 2A, *P* > 0.05). Furthermore, no significantly increased apoptotic cells in tumors were found (Figure 2B, *P* > 0.05).

We further examined if clusterin silencing affects the cispaltin sensitivity *in vivo*. A549^DDP^**/**sCLU shRNA cells were injected subcutaneously into the right flank of nude mice. As shown in Figure 2A, combined with DDP and sCLU shRNA; the tumor growth was significantly inhibited (Figure 2A, *P* < 0.05).Furthermore, the apoptotic cells in tumors were significantly increased when treatment combined with DDP and sCLU shRNA were compared with control (Figure 2B, *P* < 0.05). These findings suggest that clusterin contributes to DDP resistance in lung cancer cells in xenograft tumor models.

### Clusterin silencing *in vivo* significantly decreased pERK1/2 and pAKT

**Figure 3:**
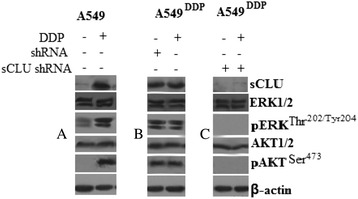
**Expression of clusterin, pERK1/2, and pAKT in A549** ^**DDP**^ **tumor tissue from the mice (A, B, C).** A549, A549^DDP^, A549^DDP^ **/**sCLU shRNA, and A549^DDP^ **/**shRNA cells were injected subcutaneously into the right flank of nude mice. Three weeks later, DDP (4 mg/kg body/wt.,i.p) was administered i.v. once every 3 days. The treatments lasted for 15 days. Protein expression in the xenograft tumor was visualized with the indicated antibodies.

Western blot indicated that the expression of clusterin, pAKT, and pERK1/2 in A549 solid tumors was weak, while it was rich in the DDP-treated A549 solid tumors (Figure 3A). In the A549^DDP^ solid tumor, the expression of clusterin, pAKT, and pERK1/2 in A549 solid tumors was very rich; however, in the DDP-treated A549^DDP^ solid tumors, no apparent increase of clusterin, pAKT, and pERK1/2 expression was found (Figure 3B). In the A549^DDP^/sCLU shRNA solid tumor, the expression of clusterin, pAKT, and pERK1/2 in A549 solid tumors was very weak; furthermore, in the DDP-treated A549^DDP^/sCLU shRNA solid tumors, the clusterin, pAKT, and pERK1/2 expression was also very weak (Figure 3C).

These findings suggest that clusterin silencing inhibits DDP-induced increase of clusterin, pAKT, and pERK1/2 expression; clusterin silencing contributes to DDP sensitiveness in lung cancer cells in xenograft tumor models, and pERK1/2 and pAKT downregulation was involved in the procedure.

### Clusterin overexpression *in vivo* significantly increased pERK1/2 and pAKT expression

**Figure 4:**
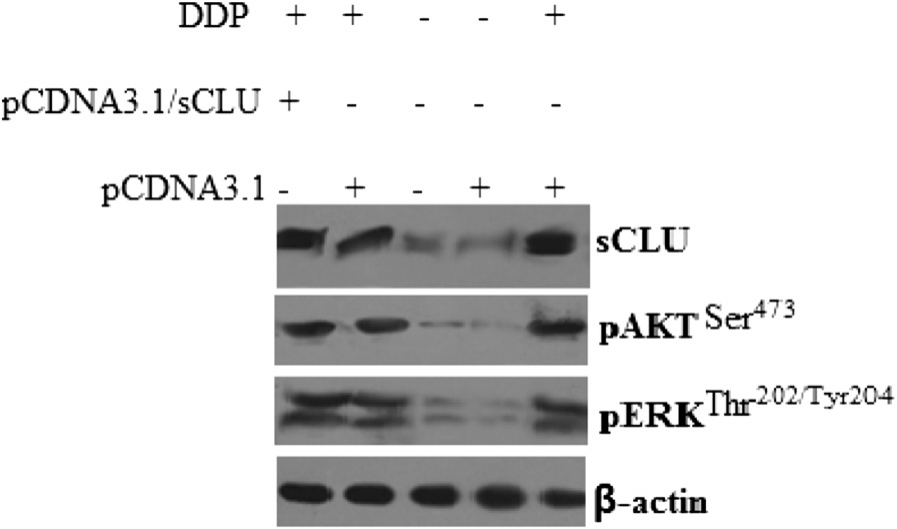
**Expression of clusterin, pERK1/2 and pAKT in A549 tumor tissue from the mice.** A549, A549**/**sCLU, and A549**/**pCDNA3.1 cells were injected subcutaneously into the right flank of nude mice. Three weeks later, DDP (4 mg/kg body/wt.,i.p) was administered i.v. once every 3 days. The treatments lasted for 15 days. Protein expression in the xenograft tumor was visualized with the indicated antibodies.

It has demonstrated above that the expression of clusterin, pAKT, and pERK1/2 in A549 solid tumors was weak (Figure 3A); however, clusterin, pAKT, and pERK1/2 expression was very rich after DDP treatment of solid tumors (Figure 4). In the DDP-treated A549**/**sCLU solid tumors, clusterin, pAKT, and pERK1/2 expression was not markedly increased than that in the control group A549**/**sCLU solid tumors (Figure 4). These findings suggest that clusterin overexpression contributes to DDP resistance in lung cancer cells in xenograft tumor models, and pERK1/2 and pAKT overexpression was involved in the procedure.

## Discussion

Despite significant advances in oncology over the last several decades, lung cancer remains highly lethal. Most patients present with advanced disease and are often inoperable at the time of diagnosis. Adjuvant cisplatin-based chemotherapy is the standard of care for completely resected high-risk stage IB and stage II NSCLC based on an approximately 5% improvement in 5-year overall survival [15]. Five promising new drugs have been shown to achieve survival rates equivalent or superior to cisplatin, and when used in combination with cisplatin or carboplatin, RRs are as high as 40% to 50%. These agents include paclitaxel, docetaxel, vinorelbine, irinotecan, and gemcitabine [16]. The limited efficacy of cytotoxic chemotherapy and radiotherapy remains a major obstacle for the treatment of patients with advanced lung cancer.

Resistance to anticancer agents is one of the primary impediments to effective cancer therapy. Chemoresistance occurs not only to clinically established therapeutic agents but also to novel targeted therapeutics. Both intrinsic and acquired mechanisms have been implicated in drug resistance, but it remains controversial which mechanisms are responsible that lead to failure of therapy in cancer patients [17]. Chemoresistance may also develop from alterations in the apoptotic machinery, secondary to increased activity of anti-apoptotic pathways or the expression of anti-apoptotic genes. Ironically, agents used to destroy malignant cells may also induce the expression of genes that mediate radiation- and chemoresistance. Survival proteins upregulated after apoptotic triggers that function to inhibit cell death include anti-apoptotic members of the bcl-2 protein family, clusterin, HSPs, and survivin [18].

Clusterin (CLU), in its cytoplasmic secretory form (sCLU), has the unique property in mediating chemoresistance to numerous unrelated anticancer agents and its presence has been observed in a variety of solid tumors and lymphoma [19]. Previous reports from our laboratory [11] have demonstrated *in vitro* that the chemotherapeutic agent DDP activated sCLU, which increased cellular DDP chemoresistance in the A549^DDP^ and sCLU transfected A549 cells via inhibition DDP-induced apoptosis; whereas sCLU knockdown induced chemosensitization in the A549 and A549^DDP^ cells via increase of DDP-induced apoptosis. Further study indicated therapies directed against sCLU have the potential to enhance the efficacy of DDP-based chemotherapy via downregulation of pAKT and pERK1/2.

The current study investigated the significance of clusterin (sCLU) silencing on DDP chemosensitivity in lung cancer cell lines and investigated the molecular mechanisms underlying the effect of sCLU silencing *in vivo*. In the first study, six groups of mice (*n* = 6) with A549^DDP^/shRNA sCLU tumors received doses of PBS (control) or cisplatin at the doses described. Tumor volumes were monitored during the study period at least twice a week. No significant decreases in tumor volume were observed with clusterin silencing and DDP monotherapies, alone. Furthermore, no significantly increased apoptotic cells in tumors were found. However, combined with DDP and sCLU shRNA, the tumor growth was significantly inhibited, and the apoptotic cells in tumors were significantly increased when treatment was combined with DDP and sCLU shRNA. Further study indicated clusterin was overexpressed in the A549^DDP^ cells. Clusterin silencing contributes to DDP sensitiveness *in vivo* via pERK1/2 and pAKT downregulation. In the next study, six groups of mice (*n* = 6) with A549/sCLU tumors received doses of PBS (control) or cisplatin at the doses described. The results showed sCLU overexpression was resistant to DDP-induced apoptosis. This effect was via pERK1/2 and pAKT upregulation.

## Conclusions

In summary, these data demonstrate that suppression of clusterin expression via siRNA transfection attenuates its anti-apoptotic effects and enhances chemosensitivity by downregulation of pERK1/2 and pAKT *in vivo*. These experimental data support the development of targeted strategies employing clusterin siRNA complementary to conventional cytotoxic therapies for advanced lung cancer.
